# Pediatric Pituitary Adenomas and Cysts: A 46-Year Population-Based Analysis

**DOI:** 10.1210/jendso/bvaf069

**Published:** 2025-04-24

**Authors:** Kaitlin Leopold, Mostafa Salama, Seema Kumar, Ana Creo, Alaa Al Nofal, Amanda Tapia, Aida Lteif

**Affiliations:** Division of Pediatric Endocrinology and Metabolism, Department of Pediatric and Adolescent Medicine, Mayo Clinic, Rochester, MN 55905, USA; Division of Pediatric Endocrinology and Metabolism, Department of Pediatric and Adolescent Medicine, Mayo Clinic, Rochester, MN 55905, USA; Division of Pediatric Endocrinology and Metabolism, Department of Pediatric and Adolescent Medicine, Mayo Clinic, Rochester, MN 55905, USA; Division of Pediatric Endocrinology and Metabolism, Department of Pediatric and Adolescent Medicine, Mayo Clinic, Rochester, MN 55905, USA; Division of Pediatric Endocrinology and Metabolism, Department of Pediatric and Adolescent Medicine, Mayo Clinic, Rochester, MN 55905, USA; Department of Quantitative Health Sciences, Division of Clinical Trials and Biostatistics, Mayo Clinic, Rochester, MN 55905, USA; Division of Pediatric Endocrinology and Metabolism, Department of Pediatric and Adolescent Medicine, Mayo Clinic, Rochester, MN 55905, USA

**Keywords:** prolactinoma, Rathke's cleft cyst, pars intermedia cyst, nonfunctioning pituitary adenoma

## Abstract

**Context:**

Pituitary adenomas and cysts are rare in pediatric patients and improved understanding can guide management recommendations.

**Objective:**

To report incidence, presentation, management, and outcomes in a pediatric population-based cohort with pituitary adenomas and cysts, and to explore the relationship between these lesions and brain magnetic resonance imaging (MRI) rates, as well as lesion size and headaches with disease progression.

**Methods:**

In this retrospective cohort study of pediatric patients (≤18 years) with incident pituitary adenomas and cysts in Olmsted County, MN, from 1976 to 2021, 234 unique patients were identified using diagnostic codes through the Rochester Epidemiology Project, with 37 confirmed cases of pituitary adenoma or cyst included. Incidence rates were calculated using census data. Descriptive statistics were used for extracted clinical data.

**Results:**

Incidence of pediatric adenomas and cysts was 2.29 cases per 100 000 person-years. Of the 37 cases, 68% were nonfunctioning adenomas or cysts, 27% were prolactinomas, and there was 1 each of growth hormone (GH)– and thyrotropin (TSH)-secreting adenomas. Median lesion diameter was 5.5 mm (IQR, 4.0-8.0). Median follow-up was 7.4 years (IQR, 4.5-15.4). Four patients had disease progression which stabilized with second-line therapy. Brain MRI rates did not correlate with lesion incidence. No clinically meaningful relationship was found between lesion size or headache and disease progression.

**Conclusion:**

Pituitary adenomas and cysts are rare in pediatric patients. Most are small, nonfunctioning, and stable on long-term follow-up. Larger studies on small nonfunctioning pituitary lesions are needed to enhance understanding of their natural history and develop long-term management recommendations.

Pituitary adenomas, Rathke's cleft cysts, and pars intermedia cysts are rare pediatric pituitary lesions that may, in a subset of patients, be associated with significant health consequences. Pituitary adenomas are a group of benign, hormonally active or nonfunctional neuroendocrine tumors arising from the adenohypophysis. Rathke's cleft cysts are non-neoplastic cystic lesions arising from the Rathke's pouch, while pars intermedia cysts are small, non-neoplastic cysts occurring at the junction of the adenohypophysis and the neurohypophysis.

Pituitary adenomas and tumors of the pituitary are estimated to represent 4% to 14.3% of all pediatric intracranial tumors [1, 2], and pituitary adenomas represent 78% of pediatric pituitary lesions [3], while pituitary cysts are reported in 1.2% to 57.7% of pediatric brain magnetic resonance imaging (MRI) scans reviewed retrospectively [4, 5]. Pituitary adenomas and cysts may be asymptomatic and are often found incidentally on imaging, or they may be symptomatic either due to mass effect from their location and size or altered hormone secretion. Management varies depending on lesion size and hormone secretory status, and consists of observation, medical management, surgery, or radiation. Treatment may result in additional endocrinopathies and complications without ensuring resolution of symptoms [6-9]. Even with treatment, large or hormone-secreting pituitary adenomas and large cysts may persist or recur and many require lifelong management.

Patient demographics, presenting symptoms, treatment, and short-term response to therapy in pediatric patients with pituitary adenomas and cysts have been reported in the setting of referral centers or surgical cohorts. In studies from tertiary care centers, most lesions were >10 mm in size and were hormone-secreting adenomas [4, 7, 10-18]. Pituitary hormone deficiencies, surgical interventions and disease recurrence were common. The length of follow-up in these studies is typically a few years, with paucity of long-term data available for patients diagnosed with pituitary adenomas and cysts as children.

Large pituitary lesions causing mass effect, hormone-secreting lesions, and lesions leading to pituitary hormone deficits require specialized care; therefore, studies from tertiary care centers likely do not represent the characteristics of pituitary adenomas and cysts at the population level and likely underrepresent small, less aggressive lesions. A geographic population-based analysis of pituitary adenomas and cysts in the pediatric age group can better describe the spectrum of disease in the population, including among patients without the need for referral to a tertiary care center. Long-term outcome data at the population level may help direct follow-up care for patients at diagnosis and may affect counseling on expected outcomes for patients at the time of diagnosis.

Our aim is to characterize this geographic population-based cohort of pediatric pituitary adenomas and cysts by analyzing incidence trends over time, clinical presentation, lesion characteristics, medical and surgical management patterns, and patient outcomes. Additionally, we examine whether the incidence of brain MRI studies in the same population correlates with the incidence of pituitary adenomas and cysts over time, exploring whether changes in MRI ordering practices influence the diagnosis rates of these lesions. Finally, we investigate the relationship between lesion size and reported headaches at presentation, as well as to disease progression, to determine if lesion size at presentation is associated with reported symptoms and long-term outcomes.

## Materials and Methods

### Study Design

This retrospective cohort study examined individuals 18 years of age or younger living in Olmsted County, Minnesota at the time of pituitary adenoma or cyst diagnosis between 1976 and 2021. Individuals were identified through the Rochester Epidemiology Project (REP), a medical record linkage system established to create a nearly all-encompassing medical record system for Olmsted County, Minnesota, which allowed for long-term population-based study of the incidence, prevalence, and outcomes of disease, as population capture exceeds 95% of Olmsted County [19].

This study was approved by the institutional review boards of the Mayo Clinic (approval no. 23-008386) and the REP Research Review Committee (approval no. 024-RRC-23).

### Eligibility and Exclusion Criteria

Pediatric patients aged ≤18 years with a coded diagnosis for pituitary adenoma or cyst were identified in the REP database using a comprehensive set of ICD-10, ICD-9, and HICDA codes (list of codes available upon request). All cases were screened by one of the authors (K.L.) to confirm the diagnosis, age at diagnosis, and county of residence at diagnosis. Any cases with an uncertain diagnosis were reviewed by the senior author (A.L.). Cases were included if there was an incident record of a pituitary adenoma or cyst on brain imaging (MRI or computed tomography [CT]). Diagnoses made by board-certified pediatric or adult endocrinologists were extracted from the medical record. In cases that underwent surgical resection, pathology reports were extracted to confirm the diagnosis. In cases where no diagnosis was made by an endocrinologist, the diagnostic impression of the radiology report was extracted and used as the diagnosis. Patients with pituitary lesions including craniopharyngiomas, gliomas, lipomas, histiocytosis, or other masses were excluded.

### Medical Record Abstraction

Comprehensive review of all available electronic and paper medical records was conducted for patients with a confirmed diagnosis of pituitary adenoma or cyst. Demographic data, including race and ethnicity, imaging indication, clinical features, symptoms at presentation, laboratory assessment, radiologic findings, visual field testing, treatment course, surgical data, pathology data, and long-term outcomes were extracted from the medical records per study protocol.

### Variable and Outcome Definitions

Race and ethnicity were reported in this study as mandated by the US National Institutes of Health (NIH) following the NIH Policy on reporting race and ethnicity data. The race of individuals included in this study was extracted from the medical record and categorized as African, Asian or Pacific Islander (which in this population included Asian, Asian Cambodian, and Asian Laotian), African American or Black, White, Other (self-reported other), or unknown (individuals for whom race was not available in the medical record). Ethnicity was extracted from the medical record and categorized as Hispanic or Latino, not Hispanic or Latino, or unknown (individuals for whom ethnicity was not available in the medical record).

The 4 long-term outcomes of disease were categorized as: stable, resolution, no known progression, or progression of disease (defined below). Outcomes were assessed as of the most recent available clinical data including laboratory evaluation, clinical documentation, and imaging findings.

The outcome was defined as stable if the patient had either no change in lesion size or decreased lesion size based on interpretation by radiologist and impression of provider on most recent pituitary-specific imaging at least 6 months from initial diagnosis, and with no clinical or biochemical evidence of disease progression as of most recent clinical data. This category included patients who underwent surgical resection with no residual disease progression or recurrence on most recent imaging and no evidence of clinical or biochemical disease progression as of most recent clinical data. An outcome of resolution was assigned when the lesion was noted to no longer be present on subsequent dedicated pituitary imaging and resolution of biochemical abnormalities if they existed. This category did not include patients who underwent surgical resection. An outcome of no known progression indicated a patient who did not have a second pituitary-specific imaging study ≥6 months after diagnosis and had no evidence of clinical or biochemical progression as of most recent available clinical data. An outcome of progression of disease indicated a patient who had evidence of increased lesion size on imaging interpretation by the radiologist and impression of provider, change in biochemical testing attributed to increasing lesion size, or clinical signs or symptoms attributed to increasing lesion size by the provider as of most recent contact with health care.

Patient visit documentation and laboratory evaluations were reviewed to determine the presence of persistent pituitary hormone deficiency following diagnosis and management. Patients with transient hormone deficiencies who no longer required treatment as of most recent clinical data were not considered to have a persistent pituitary hormone deficiency. Visual field testing was reviewed for patients for whom formal visual field testing by an optometrist or ophthalmologist was completed to assess for visual field deficits. Clinical evaluation of visual fields by physical examination was not considered to be visual field testing. Hormonal testing was interpreted as normal or abnormal using reference ranges for the patient's age and pubertal status.

Billed brain MRI studies per year in patients ≤18 years living in Olmsted County were extracted using the REP database to determine brain MRI incidence. Billed codes included all brain MRI studies independent of indication or contrast administration and were available starting in 1995 up to 2021.

### Statistical Methods

In preliminary analyses, we summarized the patients with pituitary lesions by pituitary lesion type (prolactinoma, nonfunctioning adenoma, thyroid stimulating hormone [TSH]-secreting adenoma, growth hormone [GH]-secreting adenoma, cyst [Rathke's cleft or pars intermedia], indeterminate adenoma vs cyst) and assessed distributions of demographics and baseline characteristics including imaging indication, presenting symptoms, and pubertal status. Lesion characteristics, management strategies, and patient outcomes were similarly summarized by lesion type.

Incidence was reported using standardized incidence rates for the Olmsted County census in the same year. Overall incidence of pituitary lesions (all types combined) was reported based on the number of cases per 100 000 person-years during the entire study period from 1976 to 2021. Overall brain MRI incidence rates were similarly reported for 1000 person-years from 1995 to 2021. To visually examine trends in incidence rates over time, we fit a second-degree polynomial linear model for pituitary lesion incidence and brain MRI incidence as a function of time. We further examined whether brain MRI incidence was directly associated with pituitary lesion incidence and whether that association changed over time by applying a quasi-Poisson model with log link for over dispersed count data. We estimated incidence rate ratios of pituitary lesions and 95% CIs for the main effects of brain MRI incidence and time as well as an interaction of brain MRI incidence by time.

We examined the association of lesion size at diagnosis with presenting symptoms of headaches as well as with disease progression with initial management. We presented frequency distributions by lesion size <6 mm and 6 to <10 mm and calculated relative risk of headaches and progression along with corresponding 95% CIs. The threshold of 6 mm was used to separate smaller vs larger lesions is based on Endocrine Society practice guidelines that favor routine testing for hypopituitarism and follow-up in microincidentalomas 6 mm or greater, as it is postulated these larger microincidentalomas may behave more like lesions larger than 10 mm [20]. Lesions greater than or equal to 10 mm were excluded from these 2 analyses due to the small number of lesions in this sample.

## Results

### Patient Characteristics

A total of 381 patient records, which consisted of 234 unique patients, were identified in the REP database using the comprehensive diagnostic code set for pituitary adenomas and cysts from 1976 to 2021 and screened for inclusion. Of these, 37 patients met inclusion criteria (Table 1). Excluded patients included 90 patients with a different type of central nervous system (CNS) tumor, 103 patients with no pituitary lesion or other CNS tumor, 2 patients who were 19 years or older at diagnosis of their pituitary adenoma or cyst, and 2 patients who lived outside of Olmsted County at diagnosis of their pituitary adenoma or cyst.

**Table 1.** Patient characteristics and clinical presentation of 37 pediatric patients with pituitary adenomas and cysts

	Total cases*^a,b^* (n = 37)	Prolactinoma*^a^* (n = 10)	Nonfunctioning adenoma*^a^* (n = 14)	Cyst*^a^* (Rathke's cleft or pars intermedia) (n = 8)
**Demographics**				
Age, y	16.1 (14.3-17.5)	16.6 (14.9-17.4)	16.1 (14.5-17.1)	15.2 (12.7-17.3)
Female sex	30 (81%)	9 (90%)	12 (86%)	6 (75%)
Male sex	7 (19%)	1 (10%)	2 (14%)	2 (25%)
Female:Male ratio	4.3:1	9.0:1	6.0:1	3.0:1
Race				
African	1 (3%)			
Asian or Pacific Islander	3 (8%)		2 (14%)	1 (13%)
Black or African American	2 (5%)	1 (10%)	1 (7%)	
White	29 (78%)	9 (90%)	10 (71%)	6 (75%)
Other or Unknown	3 (8%)	1 (10%)	1 (7%)	1 (13%)
More than one race reported	1 (3%)	1 (10%)		
Ethnicity				
Hispanic or Latino	2 (5%)		1 (7%)	
Not Hispanic or Latino	27 (73%)	6 (60%)	10 (71%)	7 (88%)
Unknown	8 (22%)	4 (40%)	3 (21%)	1 (13%)
**Imaging indication**				
Nonendocrine concern	8 (22%)	1 (10%)	2 (14%)	3 (38%)
Endocrine concern	29 (78%)	9 (90%)	12 (86%)	5 (63%)
**Presenting symptoms** *^c^*				
Menstrual abnormalities	12 (32%)	7 (70%)	3 (21%)	2 (25%)
Galactorrhea	5 (14%)	2 (20%)	2 (14%)	1 (13%)
Height, weight, or puberty abnormality	10 (27%)		5 (36%)	3 (38%)
Hormonal lab abnormality	10 (27%)	1 (10%)	7 (50%)	
Headache	11 (30%)	3 (30%)	5 (36%)	2 (25%)
Fatigue	3 (8%)		3 (21%)	
Nausea	1 (3%)	1 (10%)		
Vision change	1 (3%)	1 (10%)		1 (12%)
Other*^d^*	8 (22%)		5 (36%)	2 (25%)
**Pubertal status**				
Tanner II or greater	30 (81%)	10 (100%)	13 (93%)	5 (63%)
Pre-pubertal	2 (5%)			2 (25%)
Unknown	5 (14%)		1 (7%)	1 (12%)

^a^Data presented as n (percentage) or median (interquartile range).

^b^Data include all cases, including 1 thyroid stimulating (TSH)-secreting adenoma, 1 growth hormone (GH)-secreting adenoma, and 3 indeterminate lesions.

^c^Sum of category may be >100% as some patients are in more than 1 category.

^d^Other symptoms included palpitations, hirsutism, mood change, excessive sweating, orthostatic intolerance, behavioral change, anosmia, or no symptoms.

The median age of diagnosis was 16.1 years (IQR, 14.3-17.5 years). There was a female predominance for all diagnoses, with an overall female to male ratio of 4.3:1. Overall, 8% were African, African American or Black (n = 3), 8% were Asian or Pacific Islander (n = 3), and 78% of patients were White (n = 29). Also, 73% of patients were not Hispanic or Latino (n = 27) and 5% of patients were Hispanic or Latino (n = 2). Among all 37 patients, 78% of diagnoses were made from imaging obtained for a concern regarding possible endocrine dysfunction (n = 29), with the remaining made from imaging obtained for nonendocrine reasons (n = 8, 22%). Presenting symptoms were variable and dependent on underlying pathology, with most common presenting symptoms including menstrual abnormalities (n = 12, 32%) and headache (n = 11, 30%). Most patients presented with more than one symptom and the frequency of each presenting symptom is reported in Table 1. Elevated prolactin was the most common hormonal laboratory abnormality (n = 16, 43%), with 10 (63%) of these patients being diagnosed with a prolactinoma, 4 (25%) nonfunctioning adenomas, 1 cyst, and 1 indeterminate lesion.

Of the patients, 26 (70.2%) were diagnosed with adenomas and 8 patients (22.2%) with cysts. In addition, 3 lesions were categorized as indeterminate lesions, either nonfunctioning adenoma or cyst, as they had an indeterminate appearance on imaging, the patients had no hormonal abnormalities on clinical evaluation, and no confirmatory pathology was present. Of the 3 patients with indeterminate lesions, 2 were female, and all lesions were less than 10 mm in size at diagnosis. The most common diagnosis in this cohort was nonfunctioning adenoma (n = 14, 53.8% of adenomas), with prolactinoma being second most frequent (n = 10, 38.5% of adenomas). Two prolactinomas were macroadenomas with a maximum diameter ≥10 mm at diagnosis. There was 1 pathology-confirmed case of TSH-secreting macroadenoma diagnosed in an 18-year-old female patient who presented with an elevated free thyroxine level, normal TSH, and weight loss, and 1 pathology-confirmed case of GH-secreting microadenoma in a 14-year-old male patient who presented with tall stature and obesity. Most lesions (n = 32, 86%) were <10 mm in maximum dimension, and lesion characteristics by diagnosis are presented in Table 2.

**Table 2.** Lesion characteristics of 37 pituitary adenomas and cysts

	Total Cases*^a,b^* (n = 37)	Prolactinoma*^a^* (n = 10)	Nonfunctioning adenoma*^a^* (n = 14)	Cyst (Rathke's cleft or pars intermedia)*^a^* (n = 8)
Maximum dimension at presentation, mm	5.0 (4.0-7.0)	5.8 (4.3-7.5)	4.0 (3.0-5.0)	9 (8.0-11.0)
Size ≥10 mm at presentation	5 (14%)	2 (20%)	0	2 (25%)
Confirmed apoplexy	1 (3%)	1 (10%)	0	0

^a^Data presented as median (interquartile range) or number (percentage).

^b^Data include all cases, including 1 thyroid stimulating (TSH)-secreting adenoma, 1 growth hormone (GH)-secreting adenoma, and 3 indeterminate lesions.

**Figure 1.**
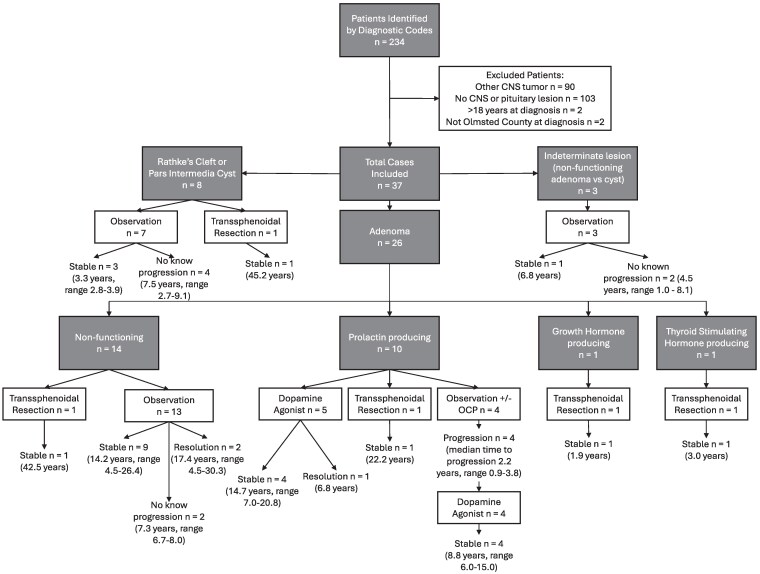
Flowchart describing the clinical management and outcomes of pediatric pituitary lesions based on diagnosis. Data presented as disease outcome (median clinical follow-up in years, range in years) or (clinical follow-up in years). Abbreviation: OCP, oral contraceptive pill.

First-line management strategies changed over time, and in this cohort, surgical intervention with transsphenoidal resection was first-line treatment in 5 patients (14%), dopamine agonist therapy with bromocriptine or cabergoline was used for 5 patients (14%), and 27 patients (73%) were managed with observation (Table 3). No patients in this cohort underwent radiation therapy for management of their pituitary adenoma or cyst. Disease progression was noted on imaging in 4 patients, all of whom had prolactin-secreting microadenomas and were initially managed with observation with or without menstrual management using a combined oral contraceptive pill. These 4 patients all started on dopamine agonist therapy after progression was noted on imaging, and clinical and imaging stability was present after dopamine agonist therapy in all 4 patients (Table 3, Fig. 1).

**Table 3.** Management and outcomes of 37 pediatric patients with pituitary adenomas and cysts

	Total cases*^a,b^* (n = 37)	Prolactinoma*^a^* (n = 10)	Nonfunctioning adenoma*^a^* (n = 14)	Cyst (Rathke's cleft or pars intermedia)*^a^* (n = 8)
**Initial management strategy**				
Observation	24 (65%)	1 (10%)	13 (93%)	7 (88%)
Dopamine agonist	5 (14%)	5 (50%)		
Transsphenoidal resection	5 (14%)	1 (10%)	1 (7%)	1 (13%)
Observation + OCP	3 (8%)	3 (30%)		
**Treatment outcomes**				
Control achieved with first-line treatment	33 (89%)	6 (60%)	14 (100%)	8 (100%)
Control achieved with second-line treatment	4 (11%)	4 (40%)		
**Disease outcome**				
Stable	26 (70%)	9 (90%)	10 (71%)	4 (50%)
Resolution	3 (8%)	1 (10%)	2 (14%)	0
No known progression	8 (22%)	0	2 (14%)	4 (50%)
**Length of follow-up, years**	7.4 (4.5-15.4)	12.3 (7.0-15.3)	11.1 (5.2-22.7)	5.7 (3.2-7.9)

Abbreviation: OCP, oral contraceptive pill.

^a^Data presented as n (percentage) or median (interquartile range).

^b^Data include all cases, including 1 thyroid stimulating (TSH)-secreting adenoma, 1 growth hormone (GH)-secreting adenoma, and 3 indeterminate lesions.

Median clinical follow-up after diagnosis for all patients was 7.4 years, and the range of follow-up was 1.0 to 45.2 years (Table 3). Thirty patients had at least one dedicated pituitary imaging study after diagnosis. The median length between diagnosis and most recent dedicated pituitary imaging was 3.5 years, with a range of 0.4 to 13.1 years. For the 7 patients without additional dedicated pituitary imaging, 2 had head CT imaging, 5.1 and 5.8 years after diagnosis for non-pituitary indications, and the remaining 5 had no additional head imaging after diagnosis.

As of the most recent available clinical data, no patients in this cohort had ongoing progression of disease with current management (Table 3). Overall, 26 patients had stable disease, 3 patients had complete resolution of their disease, and 8 patients had no known progression of disease. No patients developed a persistent pituitary endocrinopathy due to their pituitary lesion or treatment. Visual field testing was conducted for 11 patients, including 3 of the 5 patients with lesions ≥10 mm in whom visual field testing is indicated. One patient with visual field testing had visual field abnormalities. This patient had a macroadenoma and apoplexy and right-sided temporal vision loss on examination, which was his presenting symptom. This patient had normalization of visual fields with treatment. Management, length of clinical follow-up, and the clinical outcome for patients by underlying diagnosis is detailed in Table 3 and Fig. 1.

### Incidence Rates Over Time

**Figure 2.**
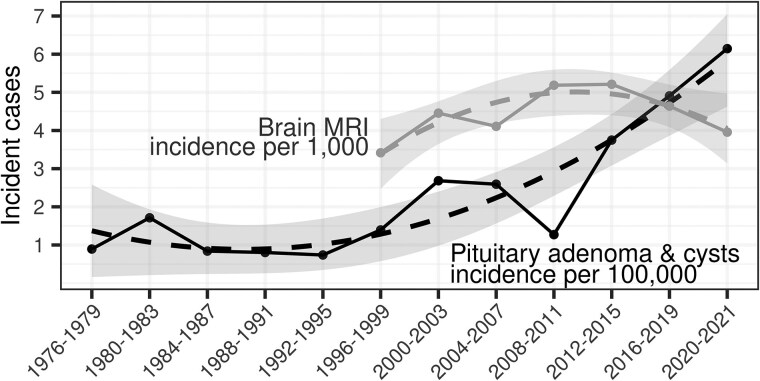
Standardized incidence rates of pituitary adenoma and cysts (per 100 000) divided into 4-year time periods (2-year time period 2020-2021) in Olmsted County, MN from 1976 to 2021, and brain MRI (per 1000) in Olmsted County, Minnesota from 1996 to 2021. The points and solid lines denote the observed incidence of pituitary adenoma and cysts (black) and brain MRI (gray). The dashed lines along with 95% confidence bands represent a smoothed linear model with second-degree polynomial over the points.

The overall incidence for pediatric adenomas and cysts from 1976 to 2021 was 2.29 cases per 100 000 person-years, with incidence increasing over time since 2000 (Fig. 2). The overall incidence per 100 000 person-years during the study period was 0.62 cases for prolactinoma, 0.87 cases for nonfunctioning adenomas, and 0.49 cases for all types of cysts.

The overall rate of performance of brain MRIs in pediatric patients living in Olmsted County from 1995 to 2021 was 443 per 100 000. From the years of 1991 to 2021, 50.2% of the brain MRIs were performed in female patients, and 49.7% in male patients. The annual rate of brain MRIs increased from 307 per 100 000 in 1995 to a peak of 577 in 2011, with a slight decline in the rate of brain MRI imaging thereafter (Fig. 2). In our regression analyses, we found that the relationship between brain MRI frequency and the incidence of pituitary lesions remained stable over time. There was no significant interaction between MRI frequency and time (incidence rate ratio [IRR] = 0.99; 95% CI: 0.69-1.39), indicating that the association between brain MRI rates and pituitary lesion incidence did not change throughout the study period. Additionally, we observed no meaningful association between the frequency of brain MRI scans and pituitary lesion incidence (IRR = 1.07, 95% CI = 0.53-2.27). However, we observed a temporal trend in pituitary lesion incidence, with the expected number of pituitary lesions increasing by a factor of 1.27 every 4 years (95% CI: 1.11, 1.45).

Regarding lesion size and its association with presenting symptom of headaches, 7 (41%) patients reported headaches among those with <6 mm lesion size, while 4 (36%) patients reported headaches among those with a 6 to <10 mm size lesion. Thus, the relative risk of headaches in patients with lesions <6 mm in size was 1.13 (95% CI: 0.43, 2.98) times that of patients with lesions 6 to <10 mm in size. Regarding lesion size and its association with disease progression with initial management, 2 (12%) patients reported progression among those with lesion size of <6 mm, while 4 (18%) patients reported progression with a 6- to 10-mm size lesion. Thus, the relative risk of disease progression in patients with lesions <6 mm in size was 0.65 (95% CI: 0.11, 3.94) times that of patients with lesions of size 6 to <10 mm. With both analyses, the relative risk is not clinically significant. (Table 4).

**Table 4.** Headaches at presentation and progression of disease for lesions based on size at diagnosis

Lesion maximum diameter	<6 mm*^a^* (n = 17)	6 to <10*^a^* (n = 11)	Relative risk (95% CI)
Headache at presentation	7 (41%)	4 (36%)	1.13 (0.43, 2.98)
Progression with initial management	2 (12%)	2 (18%)	0.65 (0.11, 3.94)

^a^Data presented as n (percentage).

## Discussion

This is the first study to characterize the incidence of pituitary adenomas and cysts in pediatric patients in a geographic population-based cohort. The overall rate of pediatric adenomas and cysts during the study period was 2.29 cases per 100 000 person-years, with the incidence of adenomas and cysts increasing over time. This trend is consistent with the trend seen in adult pituitary adenomas in Olmsted County, where pituitary adenoma incidence had increased from 1989 to 2019 [21].

This trend may represent a true increase in incidence of lesions over time, or it may be a result of changes in medical practice, technology, and documentation. Multiple factors may impact the number of diagnosed pituitary lesions over time. One cause could be an increase in incidental findings as rates of performing MRI increase. However, our model shows no relation between MRI imaging rates and pituitary lesion incidence over time, suggesting that increased utilization of brain MRIs does not explain the increase in incidence seen in this population over time. Another factor could be an increase in incidence of small lesions as imaging technology has improved over time [22]. Changes in reporting and documentation practices by radiologists and clinicians over time could result in increased rates of diagnostic coding of these lesions in patients’ medical records. Further investigation into the incidence trends of pituitary adenomas and cysts in other populations is needed to determine if this trend is isolated to Olmsted County or also occurring in other geographic populations.

In this population-based cohort of pediatric pituitary adenomas and cysts, patients tended to be adolescents with a female predominance across all diagnoses. Adolescent female predominance is reported consistently in previous studies of pituitary adenomas [7, 10, 12, 17, 23, 24], although there may be a male predominance in pre-pubertal children diagnosed with ACTH-producing adenomas [25]. Symptoms of prolactinoma may be more easily recognized in female patients due the effect on the menstrual cycle and may explain the female predominance in prolactinoma diagnosis. The rates of brain MRI in male and female patients were nearly identical and this does not explain the sex differences in diagnoses. Prior studies on pediatric Rathke's cleft and pars intermedia cysts do not consistently show a specific sex predominance [4, 26, 27].

The most commonly reported symptoms at presentation in the study were menstrual abnormalities (32%) followed by headaches (30%), and for lesions <10 mm in size, no clinically meaningful association was found between lesion size and reported symptom of headache at presentation. A limitation of this analysis was the small sample size for comparing reported headaches between the 2 groups, limiting the ability to detect clinically meaningful between the groups. A previous prospective study evaluating headache resolution in adults after transsphenoidal resection of pituitary tumors reported improvement in headaches clinically in only 53% of patients after resection without relation to tumor size [28], suggesting that many headaches in this population are not due to the tumor itself. Additionally, the overall prevalence of primary headache in children and adolescents is reported to be over 62% [29], and we hypothesize the headaches reported in these patients at presentation were unrelated to their small pituitary lesions.

The majority of patients in our study had nonfunctional lesions that were less than 10 mm in largest dimension. This finding differs from prior studies on pituitary adenomas in pediatric patients. Recent single-center cohort studies including pediatric and young adult patients show most pediatric adenomas are hormone secreting with a large percentage of macroadenomas (>10 mm in diameter) [7, 17]. In single-center studies, nonfunctioning pituitary adenomas are relatively uncommon and tend to be large, with 6 nonfunctioning adenomas of 41 cases, of which 3 were macroadenomas in one study [17] and 12 nonfunctioning adenomas of 74 cases of which all were macroadenomas in the other study [7]. Both studies report a female predominance in patients diagnosed with nonfunctioning pituitary adenomas, which is consistent with the findings of our study. This may represent a true difference in incidence of nonfunctioning adenomas between female and male patients or may represent a difference in pituitary imaging in adolescent females over males due to concerns of abnormal menses, as menstrual abnormalities were the most common presenting symptom in our cohort.

Surgical cohorts of children with pituitary adenomas studies show similar trends to single-center studies, where the vast majority of cases are hormone-secreting adenomas, a large percentage are macroadenomas, and there is a trend toward female predominance [10, 12, 23, 24].

Patients with larger adenomas causing mass effect and with hormone-secreting adenomas require medication or surgical intervention, which explains the larger proportion of large and hormone-secreting adenomas in these studies compared to our population study, as patients with smaller, nonfunctioning adenomas and cysts are likely managed and monitored by local teams and may not be evaluated at large referral or surgical centers.

The symptoms at presentation for pituitary adenomas vary based on the underlying diagnosis, with headache and menstrual dysfunction reported commonly across multiple types of adenomas [7, 17], which aligns with our study findings.

Reported incidence of incidental pituitary cysts in children is variable, with pituitary cysts reported as incidental findings in anywhere from 1.2% to 57.7% of pediatric brain MRIs reviewed retrospectively [4, 5]. Lesions tended to be small and no patients with detected lesions had pituitary dysfunction [4, 5]. Differences in rates were attributed to the improved resolution in more modern MRI images. In an autopsy review of 1000 pituitary glands, including 68 patients under the age of 20, an incidental pituitary lesion (Rathke's cleft cyst) was found in only 1 patient, an 18-year-old male individual [30].

In our study, pituitary cysts had an incidence rate of 0.49 cases per 100 000 person-years. Considering brain MRI rates in our study population, the rates of cysts are lower than would be predicted by prior studies. More than half of cysts were detected in patients undergoing pituitary imaging for an endocrine concern such as menstrual abnormalities or abnormalities in growth or puberty, where it is more likely that a small pituitary lesion will be reported or documented. Both of these factors suggest that there is either under-reporting of incidental cysts on brain MRIs by radiologists in pediatric patients, or that these lesions are not being coded by physicians, and the actual incidence is likely underestimated in our study.

In this study, abnormalities in growth or puberty were the most common presenting symptom for children diagnosed with pituitary cysts, followed by headaches and menstrual abnormalities. However, no children with pituitary cysts in this cohort had biochemical evidence of endocrine dysfunction, and their symptoms, including differences in growth and puberty, were not thought to be secondary to the pituitary cyst. This differs from prior reports of pediatric Rathke's cleft cysts where endocrinopathies, including GH deficiency and central precocious puberty are common in both small (<10 mm) and large (≥10 mm) lesions [26, 27].

Most patients in this population-based cohort had nonfunctioning lesions managed with observation alone and experienced no progression of disease. In addition, no patients developed anterior pituitary deficiencies requiring hormone replacement or persistent arginine vasopressin deficiency requiring management. In patients with hormone-secreting lesions, no patients had recurrence or progression after primary surgical resection or after management with dopamine agonist therapy. On analysis of patients with lesions less than 10 mm in size, there was no clinically significant effect of lesion size on risk of disease progression with first-line therapy, although the ability to find clinically relevant differences between groups was limited by the small sample size. Progression of disease only occurred in patients with a diagnosis of prolactinoma initially managed with observation without dopamine agonist therapy, which is the likely cause of progression rather than lesion size.

Surgical management is recommended as first-line treatment for pituitary lesions with mass effect on the brain leading to neurologic symptoms including vision loss, in hormone-secreting adenomas other than prolactinomas, and in nonfunctioning lesions resulting in a pituitary hormone deficiency [31, 32]. In our study, 5 patients underwent transsphenoidal resection for management of their pituitary lesions. The 2 patients with TSH- and GH-secreting adenomas underwent surgical resection as first-line management for their lesions. Three patients with pituitary lesions diagnosed in the late 1970s and early 1980s underwent surgical resection for first-line therapy. The diagnoses for these cases were colloid cyst, nonfunctioning pituitary adenoma, and mixed lactotroph-somatotroph (<5%) pituitary adenoma. All 3 patients had menstrual abnormalities at presentation with elevated prolactin levels on evaluation. These cases were diagnosed and treated prior to the widespread availability of dopamine agonist therapy. Bromocriptine was first approved by the Food and Drug Administration in 1978 and was labeled for use in prolactinomas in 1985 [33]. Bromocriptine became the standard medical therapy by the early 1990s [34], and the time period in which these cases were diagnosed likely impacted the choice of surgery as first-line management.

All patients who underwent surgical management had stable disease without progression or recurrence after resection.

Dopamine agonist therapy is the recommended medical therapy for patients with prolactinoma, as it is effective at decreasing prolactin levels and decreasing tumor size, although hormone replacement therapy can be considered in certain clinical scenarios [35]. In our study, all patients with prolactinomas achieved stability when treated with dopamine agonist therapy. Progression of disease only occurred in individuals with known prolactinomas being managed without dopamine agonist therapy.

Current recommendations for management of nonfunctioning pituitary adenomas in pediatric patients is surgical management with transsphenoidal resection if the patient has hormonal impairment or visual deficits, or observation with serial MRI for asymptomatic patients, with more rigorous follow-up for nonfunctioning macroadenomas than microadenomas [32, 36]. In guidelines for incidentalomas, which are defined as pituitary lesions discovered on imaging for nonendocrine indications, The Endocrine Society recommends that adults with pituitary incidentalomas undergo MRI follow-up at 6 months for macroincidentalomas, 1 year for microincidentalomas, and then every 1 to 2 years for 3 years, with a decrease in frequency after 3 years if stable [20]. Other adult guidelines recommend imaging at 1, 2, and 5 years if stable for nonfunctioning microadenomas, with reassessment thereafter for symptomatic patients [37]. French Endocrine Society guidelines for nonfunctioning pituitary incidentalomas in adults recommend no ongoing surveillance if lesions are less than 5 mm in size [38].

In this study, no patient with a nonfunctioning pituitary lesion had progression of disease with observation alone, with observational follow-up ranging from 1.0 to 30.3 years. This finding is consistent with other studies of nonfunctioning pituitary lesions. In a single-center study at Boston Children's Hospital reviewing 78 pediatric patients with incidentally discovered nonfunctioning pituitary lesions, no child had a significant increase in lesion size or experienced worsening pituitary endocrinopathy [39]. Similarly in adult data, a systematic review on adult nonfunctioning pituitary tumors showed that the risk of tumor growth and new endocrinopathies in microadenomas is low: 1.8/100 person-years for tumor growth and 0.7/100 person-years for new endocrinopathies [40]. The long-term stability of small, nonfunctioning adenomas and cysts in pediatric patients seen in this and other studies supports recommendations for less rigorous follow-up for patients with nonfunctioning microadenomas, especially for patients with lesions <5 mm.

### Strengths and Limitations

This study has several strengths. By using the Rochester Epidemiology Project (REP) medical record linkage system, cases were identified from a geographic population-based cohort which allowed for unbiased reporting of disease epidemiology. The high capture rate of the REP and the availability of data from multiple medical systems allowed for reliable longitudinal data collection on patients included. Additionally, because the geographic cohort identified by the REP coincides with our pediatric endocrinology catchment area, most patients underwent evaluation by our pediatric endocrinology practice at some point in their disease evaluation which provided valuable data for this study.

While this study does have significant strengths, there are limitations. This study relied on diagnostic codes to identify incident cases; therefore, any lesion present on imaging that was not coded in a patient record was not included. Additionally, the ability to detect small incidental lesions is dependent on imaging modality and the protocol for imaging including slice thickness, likely decreasing the detection of incidental and asymptomatic lesions in the population. Both factors likely resulted in an underestimation of asymptomatic and incidental lesions in the population. In addition, the population of Olmsted County and the majority of patients in this study were White and not Hispanic or Latino, which limits generalizability. This study relied on retrospective chart review for data collection which increases the likelihood of missing or inaccurate data from the medical record. Due to the small sample size, meaningful conclusions cannot be drawn when looking at the association between lesion size and headaches and disease progression with initial management.

## Conclusion

In conclusion, at the population level in this predominantly White cohort from Olmsted County, Minnesota, pituitary adenomas and cysts are uncommon in children and adolescents, with small, stable, nonfunctioning cysts and adenomas representing most cases. Incidence of pituitary adenomas and cysts has increased since 1995, yet outcomes are favorable with first-line management. This study provides insight into the incidence, management, and outcomes of pituitary adenomas and cysts in the first geographic population-based cohort reported in pediatric and adolescent patients. Larger studies on small nonfunctioning pituitary cysts and adenomas are needed to enhance our understanding of their natural history and to develop long-term management recommendations for affected patients.

## Data Availability

Restrictions apply to the availability of some or all data generated or analyzed during this study to preserve patient confidentiality or because they were used under license. The corresponding author will on request detail the restrictions and any conditions under which access to some data may be provided.

## Abbreviations

CT: computed tomography
GH: growth hormone
MRI: magnetic resonance imaging
REP: Rochester Epidemiology Project
TSH: thyrotropin (thyroid stimulating hormone)
